# A Modified Method in Laparoscopic Peritoneal Catheter Implantation: The Combination of Preperitoneal Tunneling and Pelvic Fixation

**DOI:** 10.1155/2013/248126

**Published:** 2013-05-15

**Authors:** Mehmet Emin Gunes, Gungor Uzum, Oguz Koc, Yiğit Duzkoylu, Meltem Kucukyilmaz, Yavuz Selim Sari, Vahit Tunalı, Sennur Kose

**Affiliations:** ^1^General Surgery Clinic, Istanbul Training and Research Hospital, Istanbul, Turkey; ^2^Nephrology Department, Istanbul Training and Research Hospital, Istanbul, Turkey

## Abstract

*Introduction*. Continuous ambulatory peritoneal dialysis (CAPD) is widely accepted for the management of end-stage renal disease (ESRD). Although not as widely used as hemodialysis, CAPD has clear advantages, especially those related to patient satisfaction and simplicity. Peritoneal dialysis (PD) catheter insertion can be accomplished by several different techniques. In this study, we aimed to evaluate our results obtained with peritoneal dialysis catheter placement by combination of pelvic fixation plus preperitoneal tunneling. *Material and Methods*. Laparoscopic peritoneal catheter implantation by combining preperitoneal tunneling and pelvic fixation methods was performed in 82 consecutive patients with end-stage renal disease. Sex, age, primary disease etiology, complications, mean duration of surgery, mean duration of hospital stay, morbidity, mortality, and catheter survival rates and surgical technique used were assessed. Analysis of catheter survival was performed using the Kaplan-Meier method. *Results*. Mean follow-up period was 28.35 ± 14.5 months (range of 13–44 months). Mean operative time was 28 ± 6 minutes, and mean duration of hospital stay was 3 ± 1 days. There were no conversions from laparoscopy to other insertion methods. None of the patients developed serious complications during surgery or the postoperative period. No infections of the exit site or subcutaneous tunnel, hemorrhagic complications, abdominal wall hernias, or extrusion of the superficial catheter cuff was detected. No mortality occurred in this series of patients. Catheter survival was found to be 92% at 3 years followup. *Conclusions*. During one-year followup, we had seven patients of migrated catheters due to separation of pelvic fixation suture from peritoneal surface, but they were reimplanted and fixated again laparoscopically with success. Over a three-year followup period, catheter survival was found to be 92%. In the literature, similar catheter survival rates without combination of the two techniques are reported. As a conclusion, although laparoscopic placement of PD catheters avoids many perioperative and early complications, as well as increasing catheter free survival period and quality of life, our results comparing to other studies in the literature indicate that different laparoscopic placement methods are still in debate, and further studies are necessary to make a more accurate decision.

## 1. Introduction

Continuous ambulatory peritoneal dialysis (CAPD) is widely accepted for the management of end-stage renal disease (ESRD) [1]. Although not as widely used as hemodialysis, CAPD has clear advantages, especially those related to patient satisfaction and simplicity. Peritoneal dialysis (PD) catheter insertion can be accomplished by any 1 of 4 techniques. These generally include open surgery, percutaneous blind insertion, peritoneoscopic (Y-Tec; Medigroup, Oswego, IL, USA), and laparoscopic techniques [2]. Lately, placement of PD catheters under flouroscopic or ultrasonographic guidance are under investigation [3, 4], but, with the evolution of laparoscopic surgery, different laparoscopic techniques have also been presented, suggesting that the technique is preferable to the open and percutaneous methods [5–8]. Open surgery and especially the percutaneous technique are associated with poor outcomes and sometimes life-threatening complications. In fact, the incidence of omental wrapping, catheter displacement, and intraabdominal complications, specifically bowel and bladder perforation, is higher with these two methods [5, 9]. In particular in patients suspected of having intra-abdominal adhesions, the application of laparoscopic surgical techniques has significantly changed our surgical approach to dialysis catheter placement, but the debate on open or laparoscopic placement of PD catheters is still ongoing [5, 8, 10, 11]. Malfunction of the peritoneal catheter is a frequent complication in peritoneal dialysis and results in catheter loss. Therefore, it is vital that the dialysis catheter tip is sited and secured accurately in the pelvis if long-term catheter function is to be achieved. This has led to improvements in laparoscopic techniques, including the preperitoneal tunneling, pelvic fixation, omentopexy, or other minimally invasive one port insertions, which aims to minimize omental wrapping and catheter dislocation [11–20].

Although each technique alone has been shown to prolong catheter survey, only Soontrapornchai's study has evaluated the combination of these two techniques in the literature [6]. We, therefore, aimed to report the results obtained with peritoneal dialysis catheter placement by combination of pelvic fixation plus preperitoneal tunneling.

## 2. Materials and Methods

Between January 2008 and June 2011, patients have undergone laparoscopic peritoneal catheter placement by combining preperitoneal tunneling and pelvic fixation (*n* = 82). A prophylactic antibiotic, cefazolin, was administered prior to the surgery. Catheter irrigation was commenced 3 days after surgery and consisted of a daily in-and-out flush with dialysate solution. Complete PD was generally commenced at the 14th day of surgery (14 ± 2).

**Figure 1:**
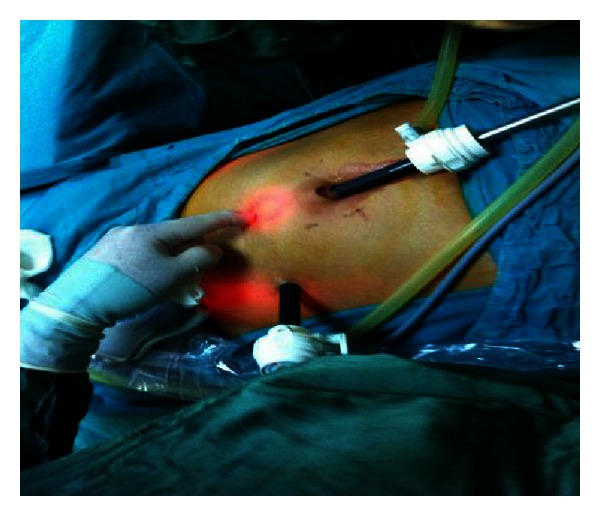
Entering sites of two ports.

**Figure 2:**
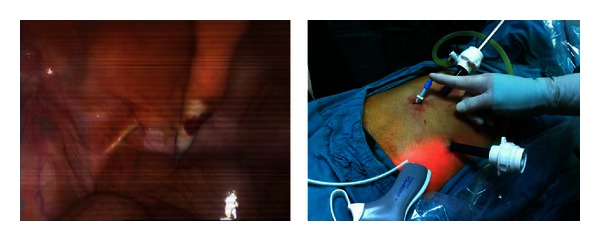
The introducer with a peel-away sheath on it which was oriented downward preperitoneally.

**Figure 3:**
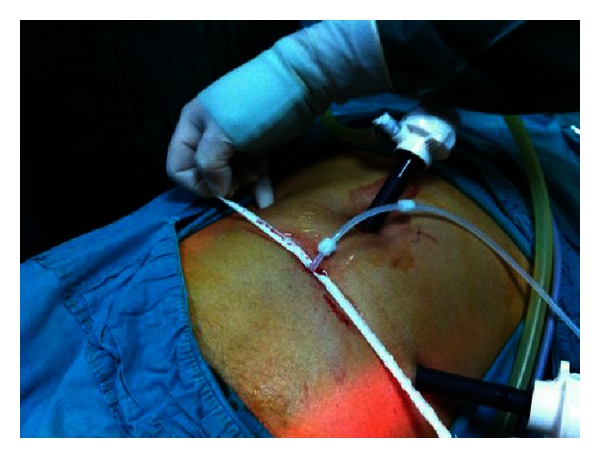
Peel-away sheet is taken out.

**Figure 4:**
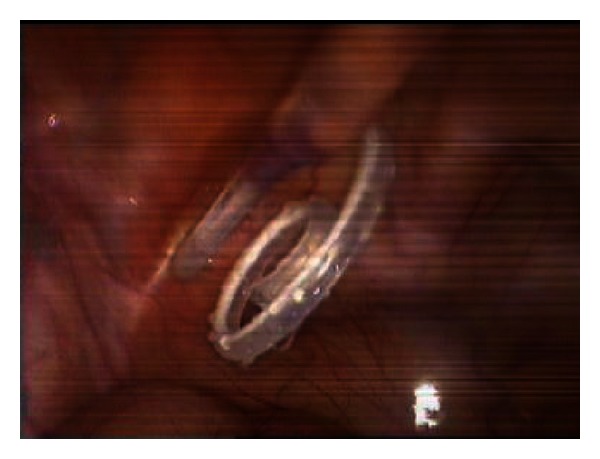
Tenckhoff catheter is introduced in the minor pelvis.

**Figure 5:**
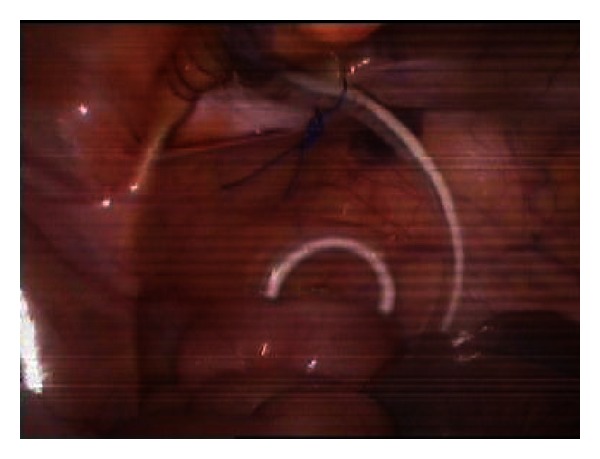
Fixation suture is performed.

**Figure 6:**
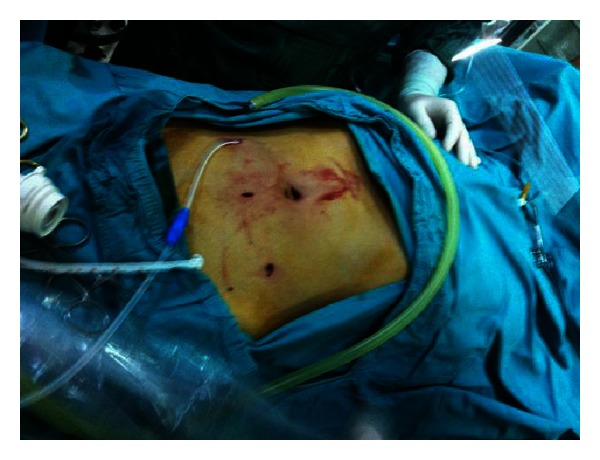
Exit site is created to the preferential of patient before the operation.

The Peritoneal Catheter kit (Quinton Instrument Company, Seattle, WA) for modified Seldinger (Littleford-Spector) technique and a 16 French Pull-Apart Sheath Introducer (Sherwood Medical Company, St. Louis, MO) are used for the catheter placement. All surgical procedures were performed under general anesthesia and by the same team experienced in laparoscopic surgery. Sex, age, primary disease etiology, complications, mean duration of surgery, mean length of hospital stay, morbidity, mortality, and catheter survival and surgical technique used were recorded. During the early postoperative period (up to 30 days), all patients were examined weekly for problems such as infection, leakage, and obstruction. Patients were then examined at 2-month intervals, and abdominal X-rays were taken to check the catheter position. The combination of laparoscopic preperitoneal tunneling and pelvic fixation used 2 ports. A telescope was inserted through a 10 mm trocar above the umbilicus after inflating the abdomen via veress needle, and a second trocar preferably 5 mm (or 10 mm in case of necessity such that Trendelenburg position was not sufficient allowing usage of a babcock forceps to retract the intestines) was placed at a point midway between the umbilicus and iliac crest on either the right or left side (Figure 1). The introducer with a peel-away sheath on it was inserted into the abdominal cavity midway between the symphysis pubis and the umbilicus vertically, and it was advanced until observing the tip of the introducer just beneath the peritoneal surface under the laparoscopic guidance but without entering into the abdominal cavity. Under laparoscopic guidance, it was advanced in the preperitoneal plane without entering into the peritoneal space by creating a tunnel at least 7-8 cm long, to the level of the bladder dome (Figure 2). At this point it was entered into the peritoneal space, and the introducer was taken out, and the curved tip of the PD catheter was introduced into the peel-away sheath (Figures 3 and 4). After placement of the curved tip of the catheter into the pelvis, it was fixed to the nearest point of the pelvic peritoneum using sliding extracorporeal knot technique by 2/0 polypropylene (Figure 5). One suture was sufficient for the fixation. The final resting position of the deep Dacron cuff was left in the rectus sheath. After testing the inflow and outflow of the catheter, a subcutaneous tunnel was created and the other tip of the catheter was taken outside which was decided preoperatively in accordance to the patient preference (Figure 6). The subcutaneous cuff was positioned at a distance of at least 2 cm from the exit wound. Port entering sites are secured by the interrupted sutures with 2/0 propylene suture material. Analysis of catheter survival was performed using the Kaplan-Meier method, with censoring of catheter loss due to death or successful transplantation.

## 3. Results

The study patients consisted of 44 (53.6%) females and 38 (46.3%) males, of average age 43 years (range of 16–76 years). Mean age and sex distribution were similar in the two groups. The primary cause of renal failure was chronic glomerulonephritis in 15 patients, diabetic nephropathy in 29, chronic pyelonephritis in 18, nephrosclerosis in 9, polycystic kidney disease in 6, and unknown origin in 5 patients. Nine patients had previously undergone intraperitoneal operations that included upper and lower abdominal incision. Although these patients had intraperitoneal adhesions, laparoscopy was performed successfully in each, and adhesiolysis was performed before insertion of the catheter. Mean duration of hospital stay is 3 ± 1 days. There were no conversion from laparoscopy in the study group. None of the patients developed serious complications during surgery or the postoperative period. No mortality occurred in this series of patients. Mean follow-up period was 28.35 ± 14.5 months (range of 13–44 months). Two patients developed an early leakage from the 5 mm port site, which was treated by application of a primary suture to the fascia of entering site. Bacterial peritonitis were seen in one patient at follow-up period and responded to antibiotic therapy. No infections of the exit site or subcutaneous tunnel, hemorrhagic complications, abdominal wall hernias, port site hernias, or extrusion of the superficial catheter cuff was detected. Eight patients (8.53%) developed catheter function disorders during three-year follow-up period, and each underwent laparoscopic revision, which restored catheter function. The cause of disfunction was catheter tip dislocation and/or omental wrapping in these patients. Catheter survival was found to be 92% at 3 years due to disfunction, death, or successful transplantation. One- and two-year catheter survival was 96% and 95%, respectively.

## 4. Discussion

Although CAPD is used more frequently than hemodialysis in European countries as a renal replacement method, the rate of CAPD is quite low in other countries. One of the primary reasons for this low rate of CAPD use is the incidence of catheter dysfunction, combined with the difficulty in performing remedial operations [18, 21]. Increasing the primary survival rate of laparoscopically implanted PD catheters will, therefore, enhance its acceptance as a replacement treatment. Among the methods used to insert PD (Tenckhoff) catheters are open surgery, blind insertion with or without radiological assistance, and laparoscopy-assisted insertion. Due to the growing worldwide acceptance of minimally invasive procedures, laparoscopic placement of peritoneal catheters is preferred over open surgery with local anesthesia, increasing catheter survival rates significantly. However, catheter function disorders can occur, generally as a result of catheter migration or omental wrapping [7, 13–21].

New techniques have been developed to avoid these complications and have led to increased survival rates [11–24]. These techniques include fixation of the omentum to the lateral abdominal wall (omentopexy) or omental excision and preperitoneal tunneling and pelvic fixation [13–27]. During the placement of PD catheters, efforts should be made to employ minimally invasive methods, using fewer ports. If the curved tip of the catheter with holes is kept in the pelvic cavity, omental wrapping and migration can be avoided. Although the results of omentopexy or omentum excision are positive, this technique necessitates greater experience in laparoscopy. Use of the preperitoneal tunneling method, while advancing the catheter in the downward position and placing it in the pelvic fossa, would eliminate the possibility of extreme migration. However, the tip of the catheter can still migrate to the right or left fossa iliaca and lead to functional disorders. Therefore, a combination of catheter fixing to the pelvic cavity at the nearest point to its tip together with preperitoneal tunneling may bring much better long-term results. The combination of omentopexy and the preperitoneal tunneling method resulted in a longer catheter survival than each method alone [13–21]. 

In addition, fixation of the tip of the catheter in the pelvic region has also been found to increase survival [6, 17]. To our knowledge, however, there have been one study of the combination of preperitoneal tunneling and pelvic fixation [6]. We, therefore, planned a study combining these techniques, omental wrapping and catheter tip migration, which cause disturbances in fluid inflow or outflow which can be minimized, to make a more clear statement about this method. We observed no significance with having compared to the literature as to the patient mortality and morbidity [6, 7, 11–17, 20–23]. Laparoscopy also permits to remedial operations such as repositioning of the catheter or dissolving the omental wrapping. During three-year followup, we had eight patients of malfunctioned catheters due to probably separation of pelvic fixation suture from peritoneal surface because they were found to be dislocated from the minor pelvis on abdominal X-rays, but they were repositioned and fixed again laparoscopically with success. Over a three-year follow-up period, catheter survival rate was found to be 92%. Maio et al. reported 91% of catheter survival rates at 3 years with their musculofascial downward incline of placement technique without pelvic fixation as a difference from our technique but in very similar fashion [24]. As a conclusion, although laparoscopic placement of PD catheters avoids many perioperative and early complications, as well as increasing catheter free survival period and quality of life, our results comparing to other studies in the literature indicate that different laparoscopic placement methods are still in debate, and further studies are necessary to make a more accurate decision [15, 18, 20, 25–27].
